# Readability of informed consent forms in clinical trials conducted in a skin research center

**Published:** 2016-07-03

**Authors:** Aniseh Samadi, Fariba Asghari

**Affiliations:** 1 MD, Pharmaceutical, Cosmeceutical, and Hygienic Evaluation Lab (DermaLab), Center for Research and Training in Skin Diseases and Leprosy, Tehran University of Medical Sciences, Tehran, Iran;; 2 Associate Professor, Medical Ethics and History of Medicine Research Center, Tehran University of Medical Sciences, Tehran, Iran.

**Keywords:** *Clinical trials*, *Informed consent*, *Readability*, *Literacy*

## Abstract

Obtaining informed consents is one of the most fundamental principles in conducting a clinical trial. In order for the consent to be informed, the patient must receive and comprehend the information appropriately. Complexity of the consent form is a common problem that has been shown to be a major barrier to comprehension for many patients. The objective of this study was to assess the readability of different templates of informed consent forms (ICFs) used in clinical trials in the Center for Research and Training in Skin Diseases and Leprosy (CRTSDL), Tehran, Iran.

This study was conducted on ICFs of 45 clinical trials of the CRTSDL affiliated with Tehran University of Medical Sciences. ICFs were tested for reading difficulty, using the readability assessments formula adjusted for the Persian language including the Flesch–Kincaid reading ease score, Flesch–Kincaid grade level, and Gunning fog index. Mean readability score of the whole text of ICFs as well as their 7 main information parts were calculated.

The mean ± SD Flesch Reading Ease score for all ICFs was 31.96 ± 5.62 that is in the difficult range. The mean ± SD grade level was calculated as 10.71 ± 1.8 (8.23–14.09) using the Flesch–Kincaid formula and 14.64 ± 1.22 (12.67–18.27) using the Gunning fog index. These results indicate that the text is expected to be understandable for an average student in the 11^th^ grade, while the ethics committee recommend grade level 8 as the standard readability level for ICFs.

The results showed that the readability scores of ICFs assessed in our study were not in the acceptable range. This means they were too complex to be understood by the general population. Ethics committees must examine the simplicity and readability of ICFs used in clinical trials.

## Introduction

Clinical trials are the gold standard for evaluating the new treatments and interventions for patients. Although preclinical studies are carefully designed, all experimental trials carry some potential risks of harm (1). To protect study participants, ethical requirements mandate that the participants be sufficiently informed about the trial before they are considered eligible to take apart in it.

 Obtaining informed consents is one of the most fundamental principles of a clinical trial and is viewed as the key to respecting participant’s autonomy (2). In fact, the value of an individual’s autonomy entitles him or her to accept or refuse any medical procedure (3). Therefore, a correct informed consent process can help patients make an autonomous decision regarding the potential harms of participation in a research (4, 5). 

The most common method of providing patients with information is the written informed consent form (ICF), while this information can be provided in many ways, such as oral discussion by the investigator and his study team, and multimedia presentations (6).

In order for the consent to be informed, the patient must receive and comprehend the information appropriately. Therefore, ICFs not only must provide the legally necessary information, but must also be prepared in a way to be completely understood by the participants (6). If patients cannot read or comprehend written materials provided to them, they will be of limited use (7, 8). Moreover, in order to improve patient comprehension of information, it is of great importance to discuss information with him/her verbally in addition to providing readable information forms. This could improve patients’ active participation in decision making and help them make wiser decisions (9).

Complexity of the consent form is a common problem that has been shown to be a major barrier to comprehension for many patients (10). The main factors of complexity include excessive length of the form, inadequate time to read the consent form, the reading level, and the format and layout of the form (11).

Low functional and health literacy is another major problem that may limit the comprehensibility of the ICFs. Health literacy is linked to literacy and entails individuals’ knowledge, motivation, and competency to access, understand, appraise, and apply health information in order to make judgments and decisions in everyday life concerning healthcare, disease prevention, and health promotion to maintain or improve quality of life (QOL) (12). Health literacy has direct correlation with patients’ capacity to participate in medical decision-making and their inclination toward participation in medical research. Low literacy may affect the decision-making process and also compliance of patients (13).

 Reading comprehension is defined as the capacity to understand the reading material content and integrate it with the basic information of the reader’s world knowledge. Clearly, it is a complicated multidimensional process that involves several factors like the integral view of the graphic material, encoding of the physical qualities of a word or letters (structural interpretation memory), and inference (14). 

Quantitative tools have been developed to evaluate the readability of written documents (15). These scoring systems give an indication of how easy a text is to read and have been used in several studies to evaluate ICFs.

The readability of ICFs has been previously assessed in several studies and different countries (1, 11, 16, 17). Most of these researches have focused on phase I trials where the potential risk ratio is especially low (18-20) and only a few studies have been dedicated to ICFs across the different phases and types of clinical trials (1). Unfortunately, most of the studies show that ICFs and other written materials for patients are prepared at levels beyond patients' literacy level (21, 22).

Since 1999 obtaining written ICFs has been a must in any interventional research involving human subjects in Iran (23). However, the readability and understandability of Iranian ICFs have not yet been evaluated.

The primary objective of this study was to assess the readability of ICFs used in clinical trials in a skin research center in Iran and determine whether there is a difference in readability between different templates of ICFs used in this research center.

## Method

*Collection of ICFs*

This study was conducted in the Center for Research and Training in Skin Diseases and Leprosy (CRTSDL) that is affiliated to Tehran University of Medical Sciences, Tehran, Iran, in 2015. This center is devoted to research and educational activities on various aspects of skin diseases and is a reference center for evaluation of pharmaceutical, cosmeceutical, and hygienic products. It is a referral center for the whole country in which more than 50 phase I to III clinical trials are conducted annually. 

ICFs were selected from among clinical trials conducted between 2008 and 2015 in field of dermatology in CRTSDL independently or in cooperation with other research centers in other cities. In total, 45 different ICFs were collected from the outpatient clinics of the secondary health-care level in Tehran and two other cities (Bam and Mashhad, Iran). We used all available ICFs, except those which were too similar in terms of methodology and experiment benefits and risks. 

 All the ICFs were approved by the Research Ethics Committee of the CRTSDL. 

Each research center has its own consent form template. Due to many studies which have been performed in cooperation with other research centers and organizations, different templates of ICFs have been used. The templates that were mainly used in the CRTSDL during this period of time have been listed below. 

*Type 1*: The template created and approved by the ethical committee of CRTSDL

*Type 2*: The proposed format of the Clinical trial Center of Tehran University of Medical Sciences (It is almost the same template proposed by the Research Ethics Committee of Tehran University of Medical Sciences) (24)

*Type 3*: The proposed format of the World Health Organization (WHO), used in Joint projects with the WHO

*Type 4*: The template used in joint projects with Mashhad University of Medical Sciences

*Type 5:* The template created by the leprosy department of the research center in cooperation with Bam Leprosy Research Center

*Type 6:* The template used in joint projects with a private dermatology outpatient clinic

*Readability assessment*

The ICFs assessed in our study included 6 main templates, depending on the 6 main cooperative research centers. Readability assessment was conducted on the templates and on the specific information sections of each trial, as well as the whole ICFs text (including the template text + information parts of the trial). The specific information sections of the trials were described as:

1. Research description
2. Voluntariness of participation
3. Participation expectation
4. Risks and benefits
5. Confidentiality
6. Principle investigators (PI) and contact information
7. Costs, compensation, and claims

In the present study, headings and sub-headings were omitted. In cases which the content within tables formed sentences, they were analyzed. In this study, three, one-hundred word sections were retrieved from near the beginning, in the middle, and near the end of documents, and the analysis was performed on this 300-word text. No computer program exists with readability formulas adjusted for the Persian language, so all calculations were performed using Microsoft Excel. Readability scores formulas were entered into the software and the scores were calculated for each ICF. Mean ± standard deviation of readability scores for each template as well as specific information sections of ICFs were also calculated using Microsoft Excel. Statistical differences in mean readability scores were tested using the univariate analysis of variances test. A *P*-value of less than 0.05 was considered as significant difference.

All the materials were in Persian, and unlike some other languages (25), there is no formula specifically designed for Persian texts.

Flesch reading-ease score (FRES) (26), Flesch-Kincaid reading grade level and age (27), and Gunning fog index (28) were chosen as the units of measure to assess readability because these indices were commonly used in medical literature (29-32). The FRES and Gunning fog index have been adjusted for Persian (33). The adjusted formulas as well as the international formula for The Flesch–Kincaid grade level are explained in appendix A.

The interpretations of readability scores have been provided in detail in table 1. 

*Flesch Reading Ease Score*

The FRES is measured using word length and sentence length. In the FRES test, higher scores indicate that the material is easier to read, but lower scores indicate that the passage is more difficult to read. 

*Flesch-Kincaid Reading Grade Level/Age level*

The Flesch–Kincaid grade level formula translates the FRES into the education grade level of the United States. 

Their results show the grade Level and age level of an individual who can understand the text. 

*Gunning Fog Index*

The Gunning fog index is a measure of text readability based upon sentence length and difficult words in a passage. The ideal score for readability with the Gunning fog index is 7 or 8. Anything above 10 is too hard for most individuals to read.

**Table 1:** Interpretation of readability scores

**Difficulty**	**Flesch reading ease score**	**Flesch-kincade grade level**	**Gunning fog index**
Very easy	91-100	4	4.9 or lower
Easy	81-90	5	5-5.9
Fairly easy	71-80	6	6-6.9
Standard	61-70	7-8	7-7.9
Fairly difficult	51-60	9-10	8-8.9
Difficult	31-50	11-14	9-9.9
Very difficult	0-30	15-16	10 or higher

## Results

In the present study, 45 ICFs of clinical trials conducted from 2008 to 2015 were investigated. 

The mean ± standard deviation (SD) of FRES for all ICFs was 31.96 ± 5.62 (range: 15.80–46.07). The mean ± SD grade level was 10.71 ± 1.8 (range: 8.23–14.09) using the Flesch–Kincaid formula and 14.64 ± 1.22 (range 12.67–18.27) using the Gunning fog index. The interpretation of readability scores has been provided in table 1. The mean scores for each group of ICFs and the readability scores of the templates are presented in table 2. 

**Figure 1:**
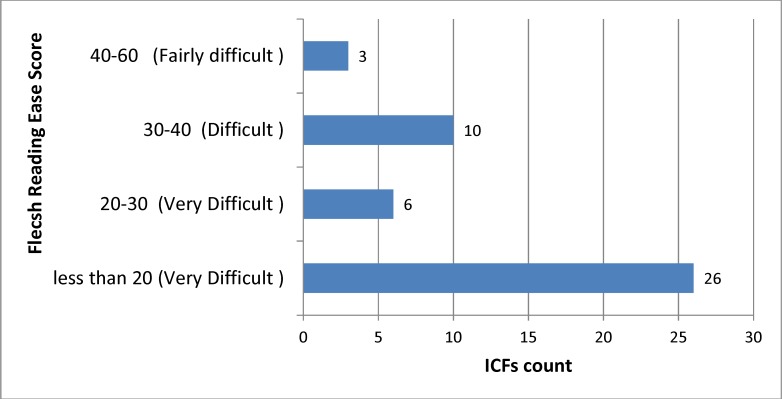
Categorical prevalence of Flesch reading ease score of informed consent forms

Readability on a grade level 8 was only found in 2.2% (n = 1) of all the ICFs assessed using the Flesch–Kincaid grade level formula and none using the Gunning fog index. Most of the studies scored less than 20 (using FRES). No ICF was found to have a score higher than 60, which indicates a standard reading level (Figure 1).

Readability of the ICFs was similar across all used templates, and no statistical differences were found between them (Table 2). 

The comparison of the templates and ICFs showed that, in some cases, the readability score of the template was beyond the standard deviation range of the ICF. This indicates that in templates type 2 and 3, there might be a significant difference in readability of the template and the related ICF (Table 2).

As was mentioned before, the readability analysis was also conducted on 7 information sections of the ICFs. Comparisons of readability scores of various sections are shown in table 3. The findings indicate that readability level was similar across all assessed information sections. However, using FRES formula, the lowest score (most difficult section of the ICFs) belonged to the section related to "risks and benefits", and the highest score (the most readable section) belonged to the "expectations from the participant". Moreover, the highest and lowest grade levels belonged to "risks and benefits" and "expectations from the participant".

**Table 2:** Distribution of informed consent forms, mean readability scores across different templates, and readability scores across template and the completed informed consent forms

**Type**	**Description**	**Number**	**Flesch reading ease**		**Flesch-Kincaid grade level**		**Flesch-Kincaid** **age level**			**Gunning fog** **index**	
			**Template**	**ICF** **(mean ± SD)**	**Template**	**ICF** **(mean ± SD)**	**Template**	**ICF** **(mean ± SD)**	**Template**		**ICF** **(mean ± SD)**
**1**	CRTSDL*	16	28.90	34.28 ± 8.82	10.51	10.59 ± 1.39	16.71 (17 years)	16.79 ± 1.39 (17 years)	13.33		14.5 ± 1.36
**2**	CTC**	9	40.81	37.96 ± 15.21	8.69	9.97 ± 1.03	14.90 (15 years)	16.18 ± 0.95 (17 years)	12.15		14.4 ± 0.93
**3**	WHO***	2	44.49	30.19 ± 7.42	7.76	11.39 ± 1.12	13.96 (14 years)	17.6 ± 1.05 (18 years)	10.54		16.51 ± 1.93
**4**	MUMS****	2	27.90	19.3 ± 10.20	11.03	13.12 ± 2.18	17.23 (18 years)	19.33 ± 2.32 (18 years)	15.15		17.87 ± 2.75
**5**	Bam	11	30.80	31.71 ± 13.12	10.33	10.89 ± 1.54	16.53 (17 years)	17.1 ± 1.90 (18 years)	14.14		14.43 ± 1.86
**6**	Private clinic	5	42.22	26.87 ± 15.31	8.49	11.49 ± 1.33	14.70 (15 years)	17.49 ± 2.90 (18 years)	13.88		13.87 ± 2.12
***P*** **-value** **across different templates**				**0.886227**		**0.499452**		**0.499452**			**0.351126**

* *Center for Research and Training in Skin Diseases and Leprosy*

** *Clinical Trial Center of Tehran University of Medical Sciences*

*** *World Health Organization*

**** *Mashhad University of Medical Sciences*

**Table 3:** Comparison of readability score of various information sections of informed consent forms (Mean with 95% confidence interval)

	**About the Trial**	**Voluntary participation**	**Expectation**	**Risks and benefits**	**Confidentiality**	**Principle investigators and contact information**	**Costs, compensation and claims**	**ANOVA** ***P*** **-value**
Flesch reading ease	31.76 ± 3.51	31.9 ± 2.51	36.15 ± 4.20	21.35 ± 4.29	29.93 ± 3.62	33.44 ± 3.43	29.22 ± 3.98	0.423
Flesch-Kincaid grade level	13.17 ± 0.99	10.38 ± 0.55	10.35 ± 0.62	16.49 ± 1.69	10.63 ± 0.41	10.29 ± 0.66	11.27 ± 0.77	0.951
Flesch-Kincaid age level	18.17 ± 0.99 (19 years)	16.59 ± 0.55 (17 years)	16.56±0.62 (17 years)	22.69 ± 0.69 (23 years)	16.83 ± 0.41 (17 years)	16.49 ± 0.66 (17 years)	17.48 ± 0.77 (18 years)	0.282
Gunning fog index	15.35 ± 0.90	14.32 ± 1.14	12.67 ± 0.78	16.81 ± 2.10	15.44 ± 0.69	12.72 ± 0.82	16.51 ± 1.83	0.981

## Discussion

*Readability Level of Informed Consent Forms*

This study assessed the readability of ICFs used in clinical trials in the CRTSDL. According to the recommendation of ethics committees, the standard readability of ICFs ranged from 5^th^-grade reading level to 10^th^-grade level (mode: 8^th^ grade) (30). Therefore, the readability of ICFs assessed in our study was not in the acceptable range (grade level 11) and they are probably too complex to be understood by the general population. Moreover, for other assessment tools, regarding the acceptable range of readability scores of ICFs (Table 1), ICFs were categorized as “difficult” to understand.

As was previously mentioned, low functional and health literacy is a problem that may limit the comprehensibility of the ICFs. About 25% of American adults are classified as having low literacy skills. American literacy surveys indicate that at least 40 million adults are functionally illiterate (left school before grade 7) and 50 million are only marginally literate (34). The data from the European Health Literacy Survey show that nearly half of the Europeans surveyed had inadequate or problematic health literacy (35). Although there has not been a definite survey about health literacy in Iran, it is estimated that the extent of this problem in Iran is even greater.

According to the report of the Statistical Center of Iran in 2013, 20 million adults in Iran are functionally illiterate and have left school in primary level (38% of adults), of whom 3.5 million are completely illiterate (36). Unfortunately, in case of ICFs assessed in our study, there was no information about the literacy level of the participants. However, the education level of the average Iranian patient in some other studies was determined as around the 5^th^ grade (37). It, therefore, appears that the readability of ICFs in this study is probably above the education level of the average patient population. 

The study was conducted in Tehran (the capital city) and two other cities of Iran (Mashhad and Bam); thus, the participants might not to be an appropriate representative of the Iranian population. However, the rate of literacy among the study participants in other cities cannot be too different.

No other ICFs readability assessment report was found in Iran to compare our finding with. Nevertheless, in one similar report on educational pamphlets in hospitals conducted in Rasht in 2013, the reported score using Flesch-Kincaid formula was almost the same we found in this study (grade level 12 in comparison with grade level 11 in this study) (38). This shows that poor level of readability is detectable in ICFs in addition to other written health related materials. 

In comparison with similar studies in other countries, in which FRESs of between 45 and 60 were obtained, the ICFs assessed in our study were less readable. Although most studies reported non-standard readability level for the ICFs of trials, their readability levels were much better than those obtained in the present study (1, 12, 34).

*Information-focused Readability Assessments*

In case of information-focused readability, the best scores belonged to information related to "expectations from the participant" and the worst to the information related to "risks and benefits". However, the difference was not statistically significant. In the review of literature, no document that assessed the readability of specific sections of the consent forms was found. This was the unique characteristic of the present study. 

There were only a few studies that focused on the length of the specific sections of ICFs. Findings showed that in this study, the length of the selected sections were noticeably short. For instance, the “risks and benefits” section consisted of 58.70 words, while in other reported papers, this section consisted of at least 318 words (1). Although the excessive length of the ICFs could be a barrier to comprehension for many patients (11), summarizing important sections like “risks and benefits” could certainly result in the participants’ lack of awareness of the potential harms.

*Different Templates of Informed Consent Forms*

As was mentioned, the assessments were performed on 6 ICF templates used in the CRTSDL.

Although the National Committee of Medical Research Ethics has worked in Iran since 2002, it has not suggested a unique template for consent forms for medical clinical trials. Therefore, research centers mostly follow the suggested format of their own regional ethics committee.

General readability assessment showed no statistical difference between the used templates; but comparison of the templates and ICFs showed significant differences in some cases. In ICF type 2 and 3 (the proposed formats of the Clinical Trial Center of Tehran University of Medical Sciences and WHO), the readability of the original templates seem significantly higher than the ICFs. This finding may indicate that, in some cases, ICFs become less readable when filled with difficult texts especially medical jargons. However, the results show that even templates developed by ethics committees are too difficult to be understood by the general population. This shows that ethics committees should pay more attention to the readability of ICFs and provide more understandable templates. 

One limitation of this study was that we did not have access to the ICF of all trials conducted during this 6 year period. Some of the PIs were not available, so their permission to access their trial documents could not be obtained. Some of the trials were conducted in cooperation with cosmeceutical and hygienic manufacturing companies and the permission of the company was required in addition to the PIs in order to access the trial documents. 

The other limitation of this study was that the readability of ICFs in one specific field (dermatology) were analyzed, while some published papers deal with ICFs from different medical fields (12, 39). 

Furthermore, there were some limitations regarding readability formulas themselves, such as lack of consideration of the influence of visual and design factors or readers’ prior knowledge and motivation (16).

It is essential that ICFs are written in clear, direct language to ensure comprehension. Words longer than three syllables, long sentences, passive sentences, and medical vocabulary could affect the readability standards. Strategies to simplify language include using short, familiar words or simple synonyms, limiting the use of polysyllabic words, and keeping sentence length less than 12 words and paragraph length less than 7 lines (40).

## Conclusion

ICFs assessed in the present study were too complex to be understood by the general population. The results of this study showed the necessity of ethics committees’ attention to readability of ICF templates prepared by different research centers. Ethics committees should also ensure that the ICFs appropriately define complex scientific concepts through simple concepts so that they can be read and comprehended by the typical subject. This assessment should be conducted more carefully in important sections like information related to "risks and benefits. Ethics committees could also use the results of this study to prepare a standard guideline for research centers to provide more understandable ICF templates.

## Appendix A

**Adjusted Formula for the Flesch Reading Ease Score (FRES) for the Persian Language:**

Flesch Reading Ease Score = 262.835 – (1.015 x ASL) – (0.846 x ASW)

ASL = Average sentence length (i.e., the number of words divided by the number of sentences)

ASW = Average number of syllables per word (i.e., the number of syllables divided by the number of words)

**The Flesch–Kincaid Grade Level Formula:**

Flesch–Kincaid Grade Level = (0.39 x ASL) + (11.8 x ASW) - 15.59

ASL = Average sentence length (i.e., the number of words divided by the number of sentences)

ASW = Average number of syllables per word (i.e., the number of syllables divided by the number of words)

**The Flesch–Kincaid Age Level Formula:**

Flesch–Kincaid Age Level = (0.39 x ASL) + (11.8 x ASW) - 10.59

ASL = Average sentence length (i.e., the number of words divided by the number of sentences)

ASW = Average number of syllables per word (i.e., the number of syllables divided by the number of words)

**The Gunning fog Grade Level Formula:**

Gunning fog grade level = 0.4 (ASL + PHW)

ASL = Average sentence length (i.e., number of words divided by the number of sentences)

PHW = Percentage of difficult words

Note: The adjustment for the Persian language was performed on the definition of difficult words not on the formula.
